# Biodegradable Polymers in Biomedical Applications: A Review—Developments, Perspectives and Future Challenges

**DOI:** 10.3390/ijms242316952

**Published:** 2023-11-29

**Authors:** Jagoda Kurowiak, Tomasz Klekiel, Romuald Będziński

**Affiliations:** Department of Biomedical Engineering, Institute of Material and Biomedical Engineering, Faculty of Mechanical Engineering, University of Zielona Góra, Licealna 9 Street, 65-417 Zielona Gora, Poland; j.kurowiak@iimb.uz.zgora.pl (J.K.); t.klekiel@iimb.uz.zgora.pl (T.K.)

**Keywords:** biodegradable polymers, biomedical applications, tissue engineering, regenerative medicine

## Abstract

Biodegradable polymers are materials that, thanks to their remarkable properties, are widely understood to be suitable for use in scientific fields such as tissue engineering and materials engineering. Due to the alarming increase in the number of diagnosed diseases and conditions, polymers are of great interest in biomedical applications especially. The use of biodegradable polymers in biomedicine is constantly expanding. The application of new techniques or the improvement of existing ones makes it possible to produce materials with desired properties, such as mechanical strength, controlled degradation time and rate and antibacterial and antimicrobial properties. In addition, these materials can take virtually unlimited shapes as a result of appropriate design. This is additionally desirable when it is necessary to develop new structures that support or restore the proper functioning of systems in the body.

## 1. Introduction

**Figure 1:**
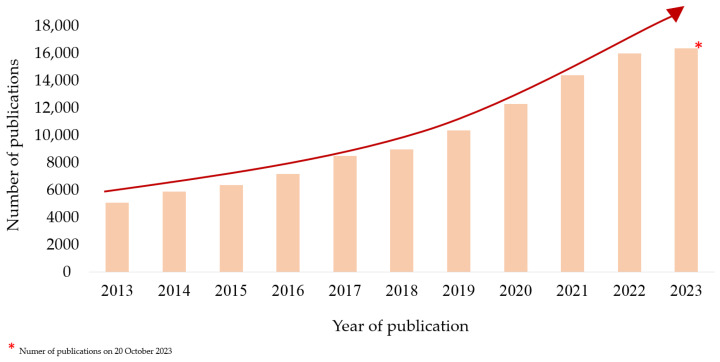
Data from the Elsevier database on the number of published scientific articles for the period 2013–2023; keyword: “polymers for biomedical applications” (in title, abstract). Source: own elaboration based on data from Elsevier database [1] as of 20 October 2023.

One of the most rapidly developing fields of science is the field related to biomaterials and their use. Intense research is leading to the development of new generations of materials, the discovery of previously unknown properties, and the manufacture of biocomposites, which can support the work of medical scientists in treatment, diagnosis and tissue regeneration even more than before. In recent years, there has been a surge in interest in polymeric materials, especially those that are biodegradable. There is growing confidence in polymers of both natural and synthetic origins. The high concentration of attention on this issue has led to a significant influx of research and has increased our access to new scientific reports. The use of biodegradable polymers is growing every year. This is confirmed by the number of emerging publications reporting on ongoing research. According to the Elsevier database (data as of 20 October 2023), the number of publications containing information on the manufacture, properties and applicability of so-called “polymers for biomedical applications” is significant, as shown in the graph in Figure 1. The use of biomaterials, nanostructures or scaffolds is currently one of the most popular issues being developed in medicine and healthcare.

As a preliminary statement, it is worth recalling the definition of a biomaterial. A biomaterial “is designed to coexist with biological systems, intended to treat, diagnose, correct or replace partially or completely a tissue, organ or perform their function in the body” [2]. Depending on the place of implantation, the disease and its advancement, medical devices made of polymers may have different contact times with tissues. The basic division of implant–organism contact includes three basic periods: short (instantaneous), lasting up to 60 min; short-term contact, up to a maximum of 30 days; long-term contact, lasting more than 30 days [2,3,4].

The criteria for polymeric materials dedicated to biomedical applications are quite stringent and are primarily driven by the safety of their future users—patients. They have been standardized and included in the ISO 10993 standard [5]. The criteria include proper material selection, manufacturing process, sterilization and effects in and on the body. All biomaterials applied to the environment of the living organism must pass a series of biocompatibility tests. Implants, which are scaffolds that have direct contact with blood, tissues, membranes or skin, are subjected to the following tests: cytotoxicity, blood compatibility, carcinogenicity, biodegradation, sensitization and reactivity with cells [2].

**Figure 2:**
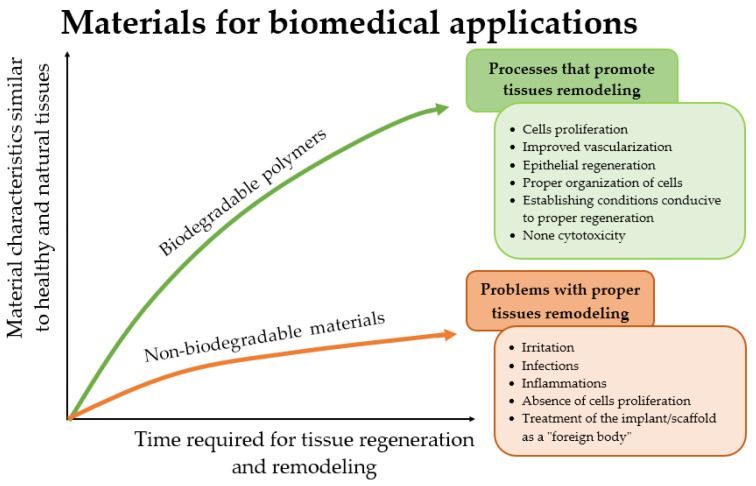
Diagram illustrating the advantages of using biodegradable materials compared to the use of solid materials in biomedical applications. Source: own compilation based on [9].

The advantages of biodegradable polymers over solid materials primarily comprise medical/clinical, financial and psychological benefits. Compared to implants made of metallic materials, for example, they do not require surgical re-intervention to remove them from the body [6,7,8]. Biodegradable polymers, both natural and synthetic, are more biocompatible than solid materials, which is more conducive to tissue regeneration. Figure 2 presents a diagram illustrating the advantage of biodegradable polymers over non-biodegradable materials, taking into account the basic advantages.

The approval of a biomaterial based on natural or synthetic polymers for internal use is preceded by detailed in vitro tests. These tests help select biomaterials with the best biocompatibility and hemocompatibility for in vivo conditions [2].

**Figure 3:**
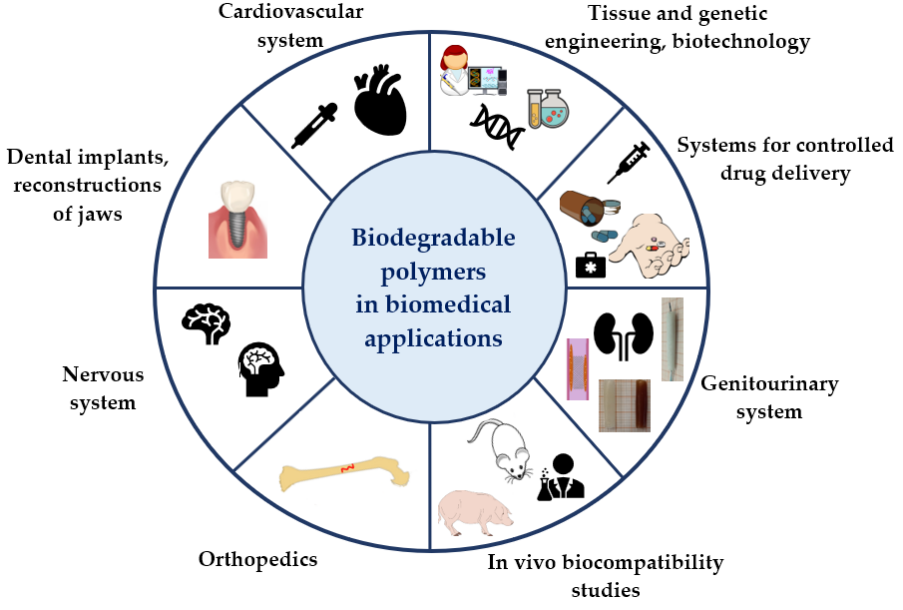
Application of biodegradable polymers in biomedical. Source: own compilation; diagram includes vector graphics (Creative Commons license).

Biodegradable polymers are widely used (Figure 3) in biomedical applications: tissue engineering and regenerative medicine [10,11,12], urology [13,14,15], controlled drug delivery systems [16,17,18], cardiac surgery [19,20], dentistry [21,22], orthopedics [23,24,25,26] and many others.

In this article, the authors attempted to characterize selected biodegradable polymeric materials of natural and synthetic origins. Then, the methods of producing these materials and data on their frequency of use are presented and described. Important factors that should not be forgotten are the criteria and requirements for biodegradable materials. Their understanding and subsequent applications can determine the success or failure of a given medical device. The manuscript also provides an overview of the applications of these materials in specific areas of biomedicine. The content presented in the article constitutes a selected and small part of a very large amount of research that is available around the world.

## 2. Biomaterials Based on Polymers

**Figure 4:**
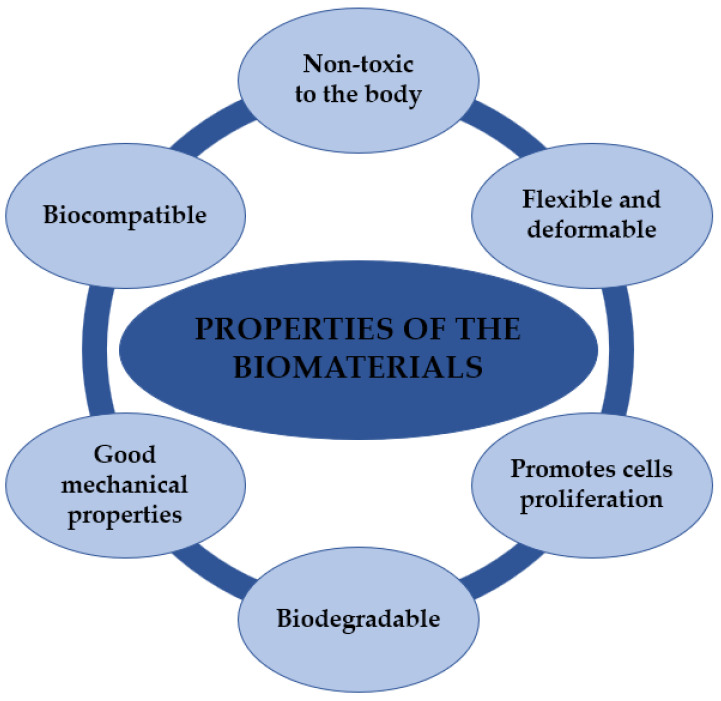
Properties of biomaterials based on natural and synthetic polymers. Source: own study.

Polymeric materials may be divided into natural and synthetic. Although they differ in origin, their functions are similar. Due to their applications in biomedicine, these materials should be characterized by certain properties. The basic characteristics of these materials are shown in Figure 4.

Polymer materials are selected individually depending on the application site. The choice of a given polymer is most often determined by its mechanical, material and biological parameters. Due to the diversity of these materials, the following subsections characterize selected polymers from the natural and synthetic groups.

### 2.1. Natural Polymers

Polymers of natural origin are of great interest in this study. Under natural conditions, they are produced by plants, animals or microorganisms. Their popularity is a result of their fairly easy availability, the low costs associated with production and their biocompatibility with living tissues [27,28]. Additionally, natural polymers are able to restore or maintain natural biological conditions, restoring function and providing structural support for the extracellular matrix (ECM) [29,30]. These are important features that support healthy, functional interactions between tissues and implanted polymers. Stimulation of cell growth and differentiation processes promotes tissue regeneration. Despite their many advantages, it is also necessary to mention the disadvantages of natural polymers. Due to the origin and low stability of the chemical structure of these materials, their strength and resistance to physicochemical stimuli are quite poor and low. It is hard to produce multiple samples from natural polymers that have consistent parameters and properties. The repeatability of results for these materials is low, and fabrication technologies such as sol-gel—although simple—do not give identical results [29,31]. Natural polymers have found widespread uses in regenerative medicine as dressing materials for hard-to-heal wounds, cosmetics and systems for controlled drug release.

#### 2.1.1. Sodium Alginate

Sodium alginate is a natural building block used in the production of absorbable and bioactive hydrogels. The sodium salt of alginic acid is an anionic and hydrophilic polysaccharide, belonging to the group of natural polymers. It is extracted from brown seaweed (*Phaeophyceae*). It is composed of linear α-L-guluronic acid copolymers (G-blocks) and slightly more branched and stretched β-D-mannuronic acid copolymers (M-blocks), which are linked by a (1,4)-glycosidic bond. The arrangement of the blocks in the structure of sodium alginate can occur in different configurations: segments of GG blocks, segments of MM blocks or in the form of their alternating MG arrangement. The structure of alginate hydrogels—richer in a higher number of M blocks—is characterized by slightly higher deformability than alginate gels, which contain a predominance of G blocks in their structure [32,33,34,35].

#### 2.1.2. Chitosan

Chitosan is obtained through the deacetylation of chitin. It is extracted primarily from crustaceans, such as shrimp and crab. In order to extract chitosan from chitin, it undergoes the aforementioned deacetylation process. Chitin deacetylation can be carried out in a strongly alkaline environment or subjected to enzymatic hydrolysis [36]. Chitosan is a linear binary heteropolsaccharide made of glucosamine. Its chemical structure contains β-1,4-N-acetylglucosamine bonds. Scientists appreciate chitosan for its properties and are betting on its use especially in tissue engineering as a scaffold for subcutaneous tissues. Chitosan exhibits high biocompatibility with tissues, is biodegradable, non-toxic and above all shows antimicrobial properties [37,38,39,40,41]. It is slightly less commonly used in drug delivery systems because it is difficult to solubilize in body fluids [41].

#### 2.1.3. Collagen

One of the most famous proteins in the human and animal body is collagen, which is a complex macroprotein. It is an essential structural component from the extracellular matrix (ECM) and accounts for one-third of all proteins in the body. It is found in structures such as skin, ligaments, cartilage and tendons [42,43]. Collagen is formed by fibrous proteins that consist of a large number of amino acids. It is considered a cellular scaffolding. Collagen is involved in inter-cellular communication, supports the immunity of organisms and has a very important function related to immunity and the reception of stimuli from mechanical stresses. Collagen comprises approximately 90% of the human body, mainly in the skin [44]. Collagen exhibits extremely valuable properties that are desirable in regenerative medicine and other biomedical applications. Collagen supports structural processes, cell growth, proliferation and migration. It is a biocompatible material, biodegradable in the tissue environment and shows no cytotoxicity to the body. It appears to be an ideal candidate for the rapid formation of tissue scaffolds [45].

#### 2.1.4. Gelatin

Gelatin is a natural polymer; it is a protein isolated by hydrolysis from animal collagen [42,46,47]. It is biocompatible and biodegradable. A large part of gelatin is water, so the mechanical strength of gelatin is low. To increase its elasticity, additives in the form of other polymers or organic or inorganic components are most often used [48]. Gelatin exhibits an extremely high ability to absorb liquids. Such conditions promote cell growth processes, which is the main task of regenerative medicine. Gelatin-based materials are unfortunately characterized by poor stability, being impermanent, susceptible to damage, and sensitive to changes in environmental conditions such as temperature [49]. Currently, the main challenge is to optimize the composition of gelatin hydrogels to increase their stability and mechanical properties.

### 2.2. Synthetic Polymers

#### 2.2.1. Poly(L-Lactide)—PLLA

Poly(L-Lactide) (PLLA) is a synthetic homopolymer derived from plants such as corn, for example. It is a representative of the polylactide PLA family. PLLA has a semi-crystalline structure of about 30–40%. The biggest advantage of this polymer is that it can be obtained from renewable sources. It is currently considered one of the most promising biomaterials for biomedical applications. PLLA is biodegradable, biocompatible, exhibits high mechanical strength, has very good physical and chemical properties and shows low toxicity in the body and tissue environment compared to other synthetic polymers [50,51].

#### 2.2.2. Polydioxanone—PDO

Polydioxanone is a synthetic, fully absorbable polymer based on poly(ester-ether). PDO is a polymer that is synthesized by the p-dioxanone monomer, which is a semi-crystalline (crystallinity of about 55%) and multi-unit repeatable ether-ester polymer, in which the ether group is responsible for the elasticity of the polymer chain network. Polydioxanone is fully biodegradable. It is believed that it could be a future material for biomedical applications [52,53,54,55] due to its good mechanical properties, biocompatibility, low inflammatory response and full metabolization by the body [56,57].

#### 2.2.3. Poly(Lactic-Co-Glycolic Acid)—PLGA

PLGA is a synthetic, biodegradable and biocompatible polymer that is obtained through polymerization with ring-opening of a lactide and a glycolide. It is most commonly used to develop systems for controlled drug release. The properties of PLGA can be designed in a controlled manner. They depend on the molar ratio between the lactide and glycolide and the molecular weight of the polymer [58,59]. PLGA appears to be a very good candidate for 3D structures. More and more studies are emerging that indicate that the use of PLGA in biomedicine may be promising. However, some limitations are emerging, indicating that PLGA can cause limited cell growth and adhesion; these may adversely affect proper soft or hard tissue regeneration [60].

#### 2.2.4. Polycaprolactone—PCL

PCL is a synthetic biodegradable polymer that is produced through ring-opening polymerization of the monomer ε—caprolactone. Polycaprolactone is a hydrophobic and semi-crystalline biopolymer. It exhibits good solubility and has a low melting point. Due to its properties, it is considered to be a good component of polymer blends [61]. The average degradation time of PCL is long and is about 2–4 years. However, this time is dependent on the molecular weight of the polymer. Breaking and disintegration in the ester bond chain causes the molecular weight to drop rapidly. This is a process that speaks to the mechanism of PCL degradation. In biomedicine, PCL is most often used in tissue engineering and as a component in controlled drug release systems [62].

## 3. Technologies of Manufacture

The development or selection of an existing manufacturing method is necessary to obtain polymer medical devices. Manufacturing a single-component implant is a simpler procedure than manufacturing a two- or multi-component compound. Due to the chemical structures of the polymers, combining them can sometimes cause difficulties. It is then important to thoroughly know each polymer and their physico-chemical properties, which will facilitate the selection of the material joining process. A finished medical implant with the desired geometry can be formed using a number of methods. These technologies include the sol–gel immersion method, electrospinning, 3D printing and bioprinting. These are currently the most popular methods used to develop and manufacture implants, scaffolds, stents or other components with applications in biomedicine primarily for internal use.

### 3.1. Sol-Gel Method

At this moment, it can be said that this is the oldest of the methods selected in this manuscript. It is characterized by great simplicity in use, versatility and low costs associated with equipment and equipment operation. The method is widely used in biomedical applications related to the synthesis of inorganic blends and polymers. Sol-gel technology involves the transition of a liquid colloidal solution into a compact three-dimensional structure; in short, a gel is obtained from a sol [63,64,65]. Ghedini et al. [66] developed a system for the controlled release of a drug in the form of antibiotics using the sol–gel method. The use of this technology made it possible to produce a topical delivery system that exhibits antimicrobial activity and prevents infection of hard-to-heal burn wounds [66]. Controlled release of ibuprofen in an aluminum oxide nanocomposite was proposed by Tarlani et al. [67]. The method of sol–gel in their research was also used by Paramita et al. [68], who aimed to develop a nano-bio-glass to promote bone tissue regeneration. The zinc-doped bio-glass nanoparticles were subjected to physical–chemical characterization and biological responses. As the authors indicate, the obtained results are promising. The use of sol–gel synthesis made it possible to develop and produce nano-bio-glass/Zn, which can be a scaffold for biomolecules, is cytologically compatible, promotes cell proliferation and osteogenic differentiation [68]. Use of this technology made it possible to produce a topical delivery system that exhibits antimicrobial activity and prevents infection of hard-to-heal burn wounds [66]. The research goal of Foroutan et al. [69] was to obtain mesoporous structures that could potentially be a good solution in the form of controlled therapeutic ion delivery systems to support bone tissue regeneration [69].

### 3.2. Electrospinning

The past decade shows that work on developing and manufacturing nanofibers is of great interest to researchers. Electrospinning is a technology that enables the development of this field. It involves the production of nano- or microfibers through a process using the electrohydrodynamic principle [70]. In the simplest terms, a strong electric field acts on a liquid polymer (solution, emulsion), which generates the formation of a polymer jet [71,72,73]. The main advantages of electrospinning are in its high efficiency, relatively easy operation of the equipment and high cost-effectiveness. Structures produced by electrospinning have a small diameter, are easily modified, have a large specific surface area, and can be shaped into various shapes [74]. Materials produced by this technology are successfully used in medicine as scaffolds, implants, or surfaces that promote the regeneration of pathologically altered tissues. Zhang et al. [75] used electrospinning to fabricate multilayered polylactide (PLA) nanofibers with the drug cisplatin. The developed matrix was designed for prolonged release of the therapeutic substance on the surgical cut, preventing local recurrence of cancer after surgical resection. The study was conducted on a mouse model. The authors indicate that the proposed solution delays cancer recurrence, further extends life span and shows less toxicity than previously used solutions [75]. Kai et al. [76] developed flexible fibers consisting of polycaprolactone (PCL) and polydimethylsiloxane (PDMS) for bone tissue engineering applications. The authors’ proposed flexible PCL/PDMS shape-memory fibers showed high biocompatibility in in vitro studies, promoted osteoblast proliferation and increased expression of biomineralization [76]. Alharbi et al. [77] undertook the development and fabrication of PLA/PVA nanofibers by coaxial electrospinning with a target application in tissue engineering. The formed nanofibers consisting of PLA core and PVA shell had very good hydrophilic properties, good mechanical properties and were cytologically compatible, which successfully enables their use for tissue regeneration [77]. Electro-spun nanofibers can also be successfully used in skin tissue engineering for regeneration [78]. Koosha et al. developed chitosan- and PVA-based electro-spun nanofibers, which they further reinforced with halloysite nanotubes. The results show that the proposed solution exhibits high biocompatibility, the developed structure is biologically compatible and the addition of halloysite nanotubes significantly improved the mechanical strength of the nanocomposite [78].

### 3.3. Three-Dimensional (3D) Printing

The incremental manufacturing method, otherwise known as 3D printing, is advancing the scientific world; 3D technologies involve the deposition of layer upon layer of material. The end result is any designed three-dimensional shape [79]. Developed in special software, 3D geometric shapes based on special codes are then uploaded to the apparatus. Several types of this technology can be distinguished: selective laser sintering (SLS), stereolithography (SLA), inkjet printing or fused deposition (FDM). One of the most popular methods is FDM. The main branches of application for this technology are the manufacture of pharmaceutical products, scaffolds for tissue engineering, structures for tissue regeneration and implants for internal use [80]. Distler et al. [81] developed and fabricated using the FDM method composite fiber scaffolds based on PLA polymer and BG bioactive glass. The scaffolds were characterized by bioactivity and cytocompatibility. The pores present in the 3D structure promoted osteo-induction, providing excellent conditions for osteoblast differentiation and ingrowth of bone-forming cells located in adjacent structures. The authors declare, based on their study, that the proposed 3D scaffolding with PLA/BG can be used in bone tissue engineering [81]. A very interesting study was presented by N’Gatta et al. [82]. The researchers produced nanocrystal-based cellulose composites using 3D printing. The developed biomimetic scaffolds made of PLA with the addition of cellulose anti-crystallose (CNC) extracted from *Ficus thonningii* showed good mechanical properties; these were found to be non-toxic to the body and biocompatible with bone cells. The proposed structural solution appears to be a good treatment method for tissue engineering as well as regenerative medicine [82]. Guerra et al. [83] developed PLA- and PCL-based cardiac stents produced using FDM printing technology. The manufactured stents were tubular in shape. Their physicochemical properties were analyzed, including degradation time, mechanical properties, and whether the stent exhibits and supports cell proliferation ability. The authors claim that the results obtained for the proposed design solution, the selected materials and the method of their fabrication could be a successful treatment for cardiovascular disease [83]. The three-dimensional scaffolds based on PCL, PLGA and hydroxyapatite for bone tissue regeneration were proposed by Ma et al. [84]. The authors suggest that the combination of these materials can positively affect the mechanical and biological properties of the printed structure. The scaffolds were subjected to mechanical and cellular tests in the presence of the bone marrow mesenchymal stem cells of a mouse model. The results indicate that the addition of PCL improves the mechanical strength of the scaffold; PLGA promotes cell proliferation and adhesion to the scaffold; hydroxyapatite enhances bone cell formation processes [84].

### 3.4. Bioprinting

Three-dimensional bioprinting is the youngest and the most personalized method used in biomedical applications. The combination of biomaterials science, tissue and organ anatomy and design and bioprinting has revolutionized the medical world. Three-dimensional bioprinting technology aims to enable the personalized creation of implants, replacement organs or tissues for patients. The advantage of bioprinting over other technologies is primarily their ability to produce structures consisting of biomaterials and cells simultaneously. Such scaffolds are almost identical to those in the body. They exhibit similar mechanical, biological and structural properties, making them functional analogs of healthy organs [85]. Given the growing number of patients with organ problems, the high number of planned transplant procedures and, at the same time, major problems with the availability of transplanted organs or tissues, bioprinting is an extremely valuable technology and the future for medicine.

Three-dimensional bioprinting can be divided into four basic techniques: inkjet bioprinting, laser-assisted bioprinting, pressure-assisted bioprinting and stereolithography. Using printers that work on what is known as “bioprinting” involves the direct deposition of very small droplets on special cell culture dishes or hydrogel structures. It is the most popular technique in 3D bioprinting and is also the cheapest. Laser-assisted bioprinting works on the principle of using a laser energy source to directly deposit material onto a substrate. The components produced by this technique can take on different sizes (from pico- to nanoscale). The variability in these parameters depends on, among other things, the biological properties of the biomaterials used, their rheology, their printing parameters and the complexity of the geometry of the printed part. Bioprinting using pressure movements of a piston or screw works by forcing biomaterials (e.g., polymers, solutions) through a nozzle onto a stationary substrate. The biomaterial extruded from the needle, usually on a microscale, is applied layer by layer to form the target 3D structure. Stereolithography, in which the liquid material, usually resin, takes a compact and solid form is based on the action of light. The advantage of stereolithography over other techniques is that it allows parts to be produced with very high accuracy [86,87,88]. The popularity of using bioprinting in biomedicine is growing every year. Wierzbicka et al. [89] conducted a study to develop and optimize bioprinting parameters for hydrogel materials based on sodium alginate and gelatin. The goal of the tests was to obtain a testing protocol and results that would ensure the best viability of osteoblast-like cells [89]. Three-dimensional hyper-elastic bone scaffolds with bacterio-static properties dedicated to specific bone defects were proposed by Shokouhimehr et al. [90]. Porous scaffolds with iron oxide nanoparticles have been tested in vitro and in vivo in an animal model of a rat with significant bone loss in the femur. The authors indicated that their proposed solution could be used in the regenerative treatment of bone tissues with a concomitant reduction in the risk of infection and contamination at the treated site [90]. Kim et al. [91] used bioprinting to produce a 3D design to treat large skeletal muscle defects. The developed implant had high structural integrity and promoted muscle cell proliferation in the process of tissue regeneration [91]. The ability to create personalized 3D constructs that are structurally similar and, above all, able to mimic tissues and organs is undoubtedly the future for medical development in generations to come.

## 4. Requirements for Polymers in Biomedical Applications

**Figure 5:**
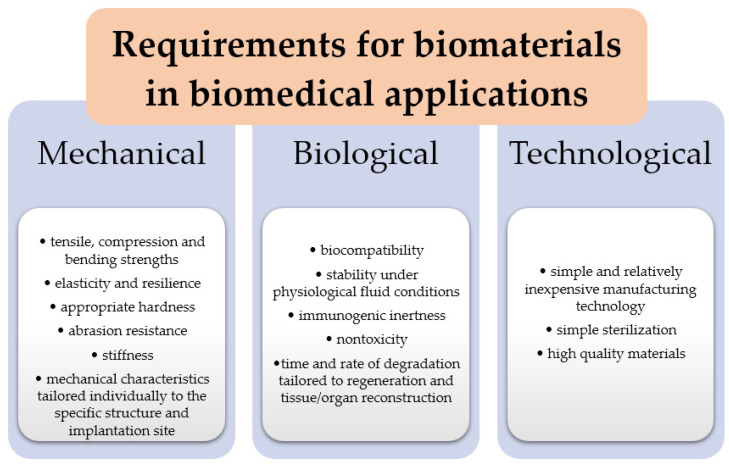
Requirements for biomaterials in biomedical application. Source: own study.

All polymeric materials that are used for internal use must meet certain—sometimes even stringent—requirements. Depending on where the biomaterial is applied, the requirements may vary somewhat. Biomaterials, in this case biopolymers, should be characterized by certain properties (Figure 5) that, firstly, will enable them to fulfill the required functions, and secondly, will not change under the influence of interaction with the body and the prevailing variable conditions in it, such as temperature, pressure, antigens or the action of X-rays or magnetic fields. All implanted implants cannot cause genetic changes, nor can they react with blood, which would lead to changes in its composition. Biodegradable polymers must not break down into products that are harmful to the body. They should not cause inflammation or infection or induce immunogenic reactions. The degradation time of polymers should be matched and sufficient for the time required for tissue and organ regeneration and reconstruction. The larger the polymer chain of the material, the longer the degradation time of the material will be [92,93].

The scientific world is still unable to cope with eliminating the body’s negative response to implants or medical devices. Very often, the body treats the implant as a foreign body that it wants expel. Work on increasing the biocompatibility of materials, their careful and specialized selection, the selection of appropriate manufacturing technology and the use of additional natural coatings can contribute to and promote better implant–tissue integration. In the long run, this can successfully influence the possibility of manufacturing implants that completely replace malfunctioning organs. Proper integration of the implant into the tissue environment, adapting to the processes of tissue regeneration and remodeling, can improve the functioning of the entire body [94].

## 5. Applications of Biopolymers

The use of polymers in biomedicine today is already versatile, and the continuous improvement of knowledge and appreciation of these materials has promoted their use in many applications as implants or prostheses. Both natural and synthetic polymers are used in tissue engineering, bone injury repair, urology, dermatology or neurology. The main function of the materials used is to provide a stable and temporary mechanical scaffold [95,96]. The human body consists of different types of tissues. Each type is characterized by different structural properties, building blocks and physical–chemical properties. Thus, the structure, geometry and properties of scaffolds must be properly designed to meet these requirements. Scaffolds dedicated to bone tissue must, first and foremost, be resistant. This is due to the functions of bone, which provide stability and protection. Bones contain more cells of the extracellular matrix, or collagen, providing mechanical strength. In contrast, soft tissues—muscles, tendons and ligaments—contain more elastin to provide elasticity and resilience, so scaffolds for these types of tissues should be more deformable and resilient [97].

### 5.1. Tissue Engineering

The damage that occurs to soft tissues can be caused by external mechanical factors such as cuts or more complicated ones resulting from associated diseases. In order to provide conditions conducive to tissue regeneration and reconstruction, scaffolds/structures based on natural and/or synthetic polymers are designed and manufactured. Polymeric materials most often undergo hydrolytic degradation, resulting in natural products metabolized by the body. The time and rate of polymer degradation depends on the type used and can range from a few weeks to months or even years [98]. The use of polymer scaffolds in soft tissue canal regeneration combines several elements, such as proper design and fabrication of the structure, collaboration with stem cells and processes that promote cell growth. During regeneration and reconstruction, damaged tissues require structural support that will further promote stem cell proliferation and migration [99].

### 5.2. Orthopedics—Bone Tissue Repair

The scaffolds dedicated to bone tissues should promote osteoinduction and osteointegration, provide structural support and have very good mechanical properties. Implants used in orthopedics should create temporary mechanical support for affected bone defect sites, promote proliferation and growth of new cells leading to bone tissue reconstruction, promote proper cell ingrowth and adhesion to the porous scaffold, promote osteoinduction and topically deliver therapeutic substances to accelerate regeneration [100]. In the case of scaffolds for orthopedics, it is very important to choose the right polymer. Hence, it is necessary to carefully examine the properties of a given material so that they are sufficient for the specific conditions of bone tissues. An improperly selected material, with mechanical properties that are too weak, will not be able to perform the functions necessary for the proper course of the healing process.

### 5.3. Urology

Disorders that occur in the urinary system are now a major health problem around the world. The number of diagnosed urological diseases is increasing every year. The most commonly diagnosed urological conditions include cancer, urethral strictures and ureteral obstruction. The task of urological reconstructions is to repair, regenerate and rebuild part or all of the urinary system. Previously used treatment methods using natural resources in the form of transplanted tissues from the inside of the cheek or small intestine are no longer sufficient today. Consequently, intensive research is being conducted to produce substitutes for urology. The availability of polymers for this type of localization is currently not a problem. A major challenge remains in understanding the actual conditions inside the urinary tract [13,14,15,35,52,101,102].

### 5.4. Neurology

An important system of any organism is the nervous system. Damage to the nervous system through injury or disease can disrupt the functioning of the body and, in a worst-case scenario, lead to death. The availability of current manufacturing methods with a large selection of polymeric materials makes it possible to develop such implants, which with their shape and properties will be able to support therapeutic treatment processes. Both natural and synthetic polymers are used in nerve tissue engineering, although currently the most trusted are those of natural origin. Polymer scaffolds help regulate biological signals, promote and direct axon growth, and slow or inhibit the formation of scar tissue. The future of polymers in the treatment of diseases of the central nervous system is extremely interesting and interdisciplinary, but also challenging and still not fully understood. The central nervous system is the most important system in the body, so it is important that proposed treatments using polymer scaffolds are safe and clinically tested [103,104,105].

The development of regenerative medicine, the science of biomaterials and the capabilities of medicine to perform more and more research aimed at understanding functions, disorders and their treatments. This is a very important aspect for mankind worldwide. Table 1 presents selected examples of the use of polymeric materials in medicine.

## 6. Perspectives for Further Research: Challenges and Constraints

The potential of natural and synthetic polymers in biomedical applications is very high. A lot of research has already been performed, providing us with valuable information on the structure, properties and potential functions of polymers. Scientists around the world are successfully conducting research into the applicability of polymeric materials. Attempts are being made to produce hybrid polymer materials and to characterize them using innovative equipment to image them and even identify their internal structures. Another important area of research is that aimed at developing polymer coatings for non-degradable materials. Such coatings are intended to increase biotolerance and acceptance at the implantation site. Surface modifications of medical devices based on biodegradable polymers may contribute to greater effectiveness in the treatment of various diseases (urological, circulatory system and difficult-to-heal wounds). In order to safely apply the proposed solutions in medical areas, it is crucial to conduct intensive research into clinical requirements [120]. Biodegradable polymer materials used in medicine, depending on the place of implantation and purpose, will be exposed to various external stimuli: pressure, flow, temperature, variable stresses and strains and electric fields. The materials used must be resistant to these stimuli and should adapt to changing conditions. It is these variables that seem to be the most challenging to understand and solve. There is no doubt that, in recent years, there has been explosive growth, success and breakthrough in the manufactured of multifunctional biodegradable polymer materials. Further research should concern chemical modifications and the creation of hybrid polymer biocomposites that are capable of imitating and regenerating bone and/or soft tissues [121].

The achievements accounted for here, and their scales, are impressive. The importance of ongoing development of these materials for medicine and the treatment of patients is evident. Interdisciplinary research combining tissue engineering, regenerative medicine and biomaterials science is opening new avenues for future research. Despite the many successes that have already been achieved, many questions continue to arise; these will continue into the future, revealing yet-undiscovered and potentially even surprising problems and challenges. Intensive research into enhancing the biocompatibility of biomaterials and their proper biological adaptation to the tissue environment must continue. Consideration should be given to the question of improving the body’s response to implants and reducing inflammatory reactions.

## 7. Conclusions and Discussion

The present article reviewed selected polymeric materials that are used in medicine. The research topic undertaken here is broad. The authors focused their attention on only some of the polymers and their specific areas of application. The challenge remains to select appropriate polymers for specific uses. The use of polymers that are too stiff and resistant to deformation will be inappropriate for implantation in hyper-elastic tissues. However, the use of polymers that are too susceptible to deformation may, for example, fail to unblock narrowed artery channels or urethras and ureters. It is necessary to deepen our knowledge about the interactions between biopolymers and tissue cells, physiological fluids and organs. In this aspect, the ongoing cooperation of many people is important—doctors, biotechnologists and biomedical engineers.

The current developments in this topic deserve the statement that this field is a powerhouse of science. Despite the many successes, researchers still face many challenges and questions: how can we improve the design, fabrication and material selection for scaffolds that are dedicated to tissue engineering and regenerative medicine? The need for more scientific work is primarily the result of a presently aging population and the occurrence of a very large number of diagnosed diseases and conditions, including new ones, whose etiologies and origins will need to be learned and rediscovered.

## Figures and Tables

**Table 1:** Selected examples of application of polymers in biomedicine.

Polymer	Technology of Manufacturing	Applications	References
Chitosan	Chemical synthesis	Thermosensitive hydrogel for musculoskeletal tissue engineering, regeneration of cartilage and meniscus	[106]
Chitosan	Chemical synthesis	Antimicrobial dressing material based on chitosan with the addition of medicinal extracts of *S. officinalis* and *H. perforatum*	[107]
PLLA	3D Printing	Topical implant made of PLLA as a functional drug release system	[108]
PLLA	Chemical synthesis	Stabilization of bone fractures with PLLA and hydroxyapatite-based implants	[109]
PLLGA Poly(L-lactide-Co-Glycolic Acid) 85:15	3D Printing	Porous scaffolds for tissue engineering with targeted application in cartilage tissue	[110]
PLLA/chitosan/chitin	Electrospinning	Resorbable conduit for peripheral nerve regeneration: reconstruction of sciatic nerve defect and restoration of nerve function and motility	[111]
PLGA/PISEB Poly(L-lactide-co-glycolide)/poly(isosorbide sebacate)	Electrospinning	Scaffold for blood vessel regeneration	[112]
PLCL/RSSP Poly(L-lactide-co-ε-caprolactone)/recombinant spider silk protein	Electrospinning	Scaffolds for skin regeneration in the form of nanofibrous membranes	[113]
PHBV/PLGA poly(3-hydroxybutyrate-co-3-hydroxyvalerate)/poly(D,L-lactide-co-glycolide)	Additive Manufacturing (AM)	Scaffolds for bone tissue regeneration	[114]
Sodium alginate	Chemical synthesis	Alginate microsponge scaffolds as drug delivery systems for rheumatoid arthritis	[115]
Sodium Alginate/Gelatin	3D bioprinting	Tissue engineering: tissue implants	[116]
PDO Polydioxanone	3D Printing	Biodegradable urological stent for the treatment of urethral stenosis	[52]
PCL/PGC polycaprolactone/polyglecaprone	Electrospinning	Nanofibrous scaffolds for regeneration of human articular cartilage and soft bones	[117]
Chitosan/Bacterial Cellulose	Chemical synthesis	Hydrogel dressing for wound healing and tissue repair	[118]
Poly(1,4-butanediol citrate)	Chemical synthesis	Materials for wound dressings, cell culture mediums	[119]
